# Biocatalytic System Made of 3D Chitin, Silica Nanopowder and Horseradish Peroxidase for the Removal of 17α-Ethinylestradiol: Determination of Process Efficiency and Degradation Mechanism †

**DOI:** 10.3390/molecules27041354

**Published:** 2022-02-17

**Authors:** Tomasz Machałowski, Katarzyna Jankowska, Karolina Bachosz, Wojciech Smułek, Hermann Ehrlich, Ewa Kaczorek, Jakub Zdarta, Teofil Jesionowski

**Affiliations:** 1 Institute of Chemical Technology and Engineering, Faculty of Chemical Technology, Poznan University of Technology, Berdychowo 4, 60-965 Poznan, Poland; tomasz.g.machalowski@doctorate.put.poznan.pl (T.M.); katjan@kt.dtu.dk (K.J.); karolina.h.bachosz@doctorate.put.poznan.pl (K.B.); wojciech.smulek@put.poznan.pl (W.S.); ewa.kaczorek@put.poznan.pl (E.K.); 2 Process and Systems Engineering Centre (PROSYS), Department of Chemical and Biochemical Engineering, Technical University of Denmark, Søltofts Plads, Building 227, DK-2800 Kongens Lyngby, Denmark; 3 Institute of Electronic and Sensor Materials, TU Bergakademie Freiberg, Gustav-Zeuner Str. 3, 09599 Freiberg, Germany; hermann.ehrlich@esm.tu-freiberg.de; 4 Center for Advanced Technologies, Adam Mickiewicz University, Uniwersytetu Poznanskiego 10, 61-614 Poznan, Poland

**Keywords:** silica nanopowder, chitin, enzyme immobilization, horseradish peroxidase, 17α-ethinylestradiol, degradation

## Abstract

The occurrence of 17α-ethinylestradiol (EE2) in the environment and its removal have drawn special attention from the scientific community in recent years, due to its hazardous effects on human and wildlife around the world. Therefore, the aim of this study was to produce an efficient enzymatic system for the removal of EE2 from aqueous solutions. For the first time, commercial silica nanopowder and 3D fibrous chitinous scaffolds from *Aplysina fistularis* marine sponge were used as supports for horseradish peroxidase (HRP) immobilization. The effect of several process parameters onto the removal mechanism of EE2 by enzymatic conversion and adsorption of EE2 were investigated here, including system type, pH, temperature and concentrations of H_2_O_2_ and EE2. It was possible to fully remove EE2 from aqueous solutions using system SiO_2_(HRP)–chitin(HRP) over a wide investigated pH range (5–9) and temperature ranges (4–45 °C). Moreover, the most suitable process conditions have been determined at pH 7, temperature 25 °C and H_2_O_2_ and EE2 concentrations equaling 2 mM and 1 mg/L, respectively. As determined, it was possible to reuse the nanoSiO_2_(HRP)–chitin(HRP) system to obtain even 55% EE2 degradation efficiency after five consecutive catalytic cycles.

## 1. Introduction

Nowadays, increasing environmental pollution by endocrine-disrupting chemicals (EDCs) is a dangerous risk to wildlife and human health, which is caused by the constantly growing pharmaceutical and veterinary market and everyday consumption of these chemicals [1]. It was proved that EDCs mimic natural hormones and can lead to many disorders of the body’s functioning, such as blocking of receptors or limitations of metabolic processes [2,3]. In work presented by Nazari and Suja [4], it was described that EDCs may cause male’s gonad dysmorphias and embryo deformations. Further, in work by Adeel et al. [5], it was shown that there is relationship between the presence of estrogens in the environment and breast cancer incidence. What is important is that two main sources of surface water contamination by EDCs may be distinguished as follows: (i) overflow from areas fertilized with manure and (ii) flow into streams from sewage treatment plants. Unfortunately, traditional treatment methods, i.e., coagulation, sedimentation or treatment by chlorine, do not allow for effective removal of most of the EDCs. Therefore, solutions with hormones, their metabolites and pharmaceuticals at high concentrations can easily and directly reach surface waters and then lead to exposure to wildlife [4]. Thus, efficient and eco-friendly methods of EDCs removal are still being sought due to the fact that they are crucial for limitation of those compounds in natural waters.

Enzymatic degradation seems to be a very promising method for the elimination of EDCs from aqueous solutions. The special attention should be paid to oxidoreductases, especially laccases, peroxidases and tyrosinases, which can effectively oxidize phenolic compounds to produce usually less toxic products than the initial substrate [6]. Thus, hormones such as estrone, 17β-estradiol or 17α-ethinylestradiol, consisting of phenolic structures, can be successfully degraded even by small amounts of enzymes [2]. Even though the literature offers reports concerning the use of free enzymes in EDCs remediation, some authors indicate the advantages of enzyme immobilization [1,7]. Such action open industrial applicability of the enzyme-based materials, as well as to enhance their stability and activity under harsh process conditions [8]. Moreover, enzyme immobilization allow us to minimize costs, since both enzyme and supporting scaffold can be used in the many degradation cycles [1].

Among oxidoreductases, the enzyme with a high application potential is horseradish peroxidase (HRP). This biomolecule possesses two metal centers in its structure, which are ferroporphyrin—responsible for redox reaction—and two calcium ions, which stabilize the enzyme’s three-dimensional structure during oxidation processes [9]. Horseradish peroxidase can effectively catalyze the oxidation reaction of a wide range of phenolic compounds, such as dyes, bisphenols, chlorophenol or pharmaceuticals. For example, Li et al. [10] used horseradish peroxidase immobilized onto electrospun magnetic nanofibers for removal of phenol from aqueous solution, with 85% efficiency, whereas Jankowska et al. [11] applied this biomolecule, immobilized onto polyamide 6 fibers, as a tool for decolorization of textile dyes from model sea waters. However, the most interesting application of immobilized HRP could be the removal of pharmaceuticals and hormones from aqueous solutions. What is more interesting is that there are only a few scientific reports regarding the application of this oxidoreductase for removal of estrogens from waters. In one of them, Ai et al. [12] conducted an immobilization process of HRP onto Fe_3_O_4_@SiO_2_ microspheres modified by amine groups and glutaraldehyde. It was shown that 17β-estradiol was removed using such biocatalytic systems, with 80% efficiency; however, after seven consecutive catalytic cycles, the degradation efficiency equaled only 45%. Therefore, despite the relatively high removal efficiency of EDCs by immobilized HRP, there is still a need to develop new systems based on this biomolecule for more effective removal processes.

Among various enzymes’ supports, inorganic particles are widely used due to their advantageous properties [13,14,15,16]. One of the most interesting supports for enzyme immobilization is SiO_2_. This inexpensive material, due to its properties such as lack of toxicity, great chemical activity and high porosity as well as the presence of hydroxyl groups onto its surface [17,18], has been widely used for attachment of various enzymes. As was presented by Mondal et al. [19], urease immobilized by covalent binding onto a 3D silica gel, retained 100% of its catalytic activity after 30 days, whereas Libertino et al. [20] showed that glucose oxidase immobilized onto porous SiO_2_ was able to retain its activity even after 3 months of storage. Recently, more and more attention is paid on nanoSiO_2_, mainly due to its highly porous structure and high thermal and chemical stability. Moreover, it prevents biomolecules inactivation due to stabilization of the enzyme structure. It was reported that t_1/2_ of the porcine pancreatic lipase immobilized onto nanosilica was 25 days, whereas free lipase lost 50% of its activity after 2 days of storage [21]. The other interesting support for enzyme immobilization seems to be renewable, naturally pre-structured 3D chitinous scaffolds isolated from cultivated marine sponges. The unique 3D morphology of naturally formed chitinous scaffolds from marine sponges has been used numerous times in various technological and biomedical applications [22,23,24,25]. For instance, Wysokowski and co-authors synthesized advanced functional materials, such as chitin-ZrO_2_ [26], chitin-GeO_2_ [27] or chitin-(Ti,Zr)O_2_ composites [28] in terms of their photoluminescent, catalytic and photocatalytic properties. Moreover, these natural biopolymers, in the form of three-dimensional scaffolds, were used as green adsorbent of environmental pollutants [29] or regenerative medicine assays [30,31,32,33,34]. Chitin owes its versatility to the presence of numerous functional groups typical for this structural aminopolysaccharide, mainly carbonyl and hydroxyl groups [35], which provide valuable possibilities for instance for enzyme binding and offer a wide range of modification possibilities [36,37]. Recently, a microporous 3D chitinous scaffold from *Aplysina archeri* marine sponge has been successfully used for laccase immobilization and the removal of selected pharmaceuticals from an aqueous solution [8]. As observed, the removal efficiency of the tetracycline by simultaneous adsorption and catalytic conversion was determined as above 90% in various pH (4–6) and temperature (25–45 °C) from solutions at concentrations up to 1 mg/L [10]. These data indicated that 3D chitin-based biocatalytic systems might be considered as a highly effective alternative for the removal of pollutants from aqueous solutions, which was motivation for us for further development.

To improve the removal efficiency of estrogens in our research, we have focused on designing of a novel biocatalytic systems formed after various combinations of chitin and nanosilica, pristine and with immobilized horseradish peroxidase to establish practical utility of the EE2 removal system composed for removal of synthetic 17α-ethinylestradiol (EE2) from aqueous solutions. Horseradish peroxidase has been immobilized onto silica nanopowder (by adsorption) and on chitinous scaffold isolated from the *A. fistularis* marine sponge (by adsorption), and these materials were supported in the second stage of the experiments by pristine sorbents or supports with immobilized enzyme. The crucial part of the work was determination of the mechanism of estrogen removal by synergistic adsorption onto the proposed support and bioconversion. The effect of the process parameters onto the EE2 removal efficiency has been determined, including the temperature, pH, concentration of EE2 or amount of hydrogen peroxide. Obtained results clearly show the great potential of the proposed systems for the removal of estrogens and reduction in toxicity of parent mixtures.

## 2. Results and Discussion

### 2.1. Physicochemical Characterization of the EE2 Removal System

The interest in use of natural materials and oxides as carriers for enzymes immobilization is still increasing, due to their renewability, low cost, and well-developed chemical and porous structure [38]. Herein, both materials, chitin and nanosilica, were used for HRP immobilization, and the effectiveness of the immobilization process has been determined. In the case of nanosilica, immobilization was carried out using adsorption and next cross-linking using 0.1% solution of glutaraldehyde as a linker. The cover of nanosilica with the immobilized enzyme using glutaraldehyde was dictated by the fact that this compound can react with the enzyme’s amino groups, leading to formation of stable intermolecular interactions that cause an increase in the biomolecule’s stability [11,39,40]. Due to the fact that silica does not contain amine groups on its surface, our intention, based on the previously published studies [11,41], was to use glutaraldehyde to directly cross-link enzyme molecules to each other. This approach strongly reduces enzyme leaching and its mobility as well as providing more stability and additional protection against inactivation. In case of chitin, it was decided to carry out HRP immobilization process using adsorption. This natural material possesses various functional groups, such as C=O and –NH, which may facilitate enzyme-support connection [42]. It was calculated that the immobilization efficiency of HRP onto chitin equaled 10%, and it was around 37% for the system based on nanoSiO_2_. The differences in immobilization efficiency may be related to the applied immobilization method and various porosity of supports used. In the case of the nanoSiO_2_(HRP) EE2 removal system, the enzyme was immobilized by adsorption and further cross-linking onto nanoporous material, whereas HRP, immobilized onto a chitin scaffold, was attached by adsorption onto a support with interconnected macropores [29,30,43,44]. What is more, chitinous fibers are characterized by a three-dimensional open structure which maintains effective HRP enzyme immobilization.

**Figure 1:**
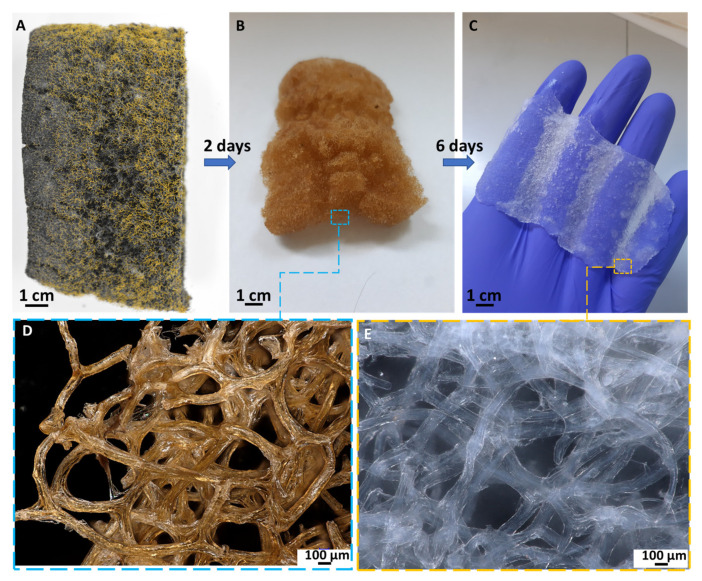
Schematic illustration of chitinous scaffold isolated from marine sponge *A. fistularis*. (**A**) Selected fragment of air-dried sponge. (**B**,**D**) The same fragment of the skeleton after 2 days of acid-base treatment. (**C**,**E**) Fully transparent and soft chitinous scaffold after isolation.

The microstructure of chitinous matrix from *A. Fistularis* marine sponge skeleton is presented in Figure 1. The typical microporous sponge morphology of the chitinous fibers-based scaffold [30,44] after isolation process can be observed. Moreover, it could be seen that the single fibers’ diameter range is 75–100 µm. It should be stated that, compared with other “chitin sources”, only marine sponges can form tube-like and structurally organized 3D chitinous skeletons. Therefore, successfully conducted isolation allows us to obtain “ready to use” scaffolds [30] with properties suitable for enzyme immobilization and for biocatalytic applications. The first step in the determination of a carrier’s morphological changes was making observations using a digital microscope (see Supplementary Materials Figure S1). The visible changes in the color of the carriers were detected after the HRP immobilization, which suggests successful enzyme deposition.

**Figure 2:**
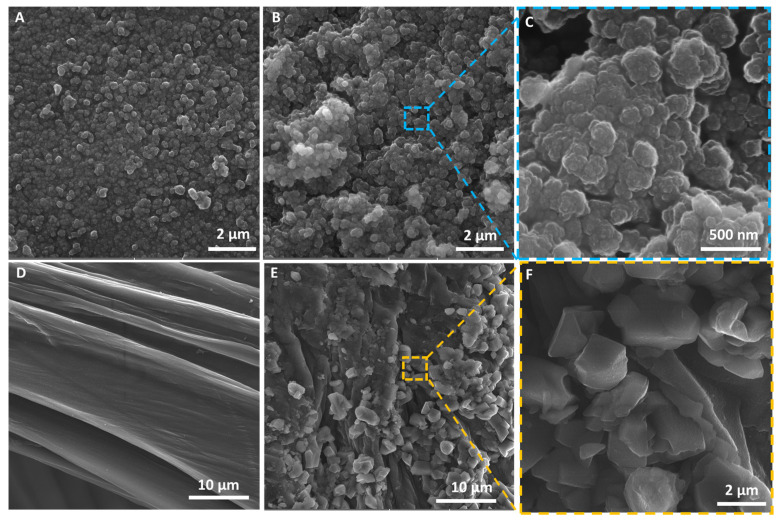
SEM images of (**A**) silica nanopowder before immobilization and (**B**,**C**) after immobilization of HRP. Chitinous scaffold isolated from *A. fistularis* (**D**) before immobilization and (**E**,**F**) after enzyme immobilization.

SEM photographs were made before and after immobilization of horseradish peroxidase for both the silica nanopowder and chitin-based scaffold (Figure 2). Based on the images presented, it can be seen that silica nanopowder before enzyme immobilization (Figure 2A) is characterized by homogeneous particles with smooth texture, whereas after enzyme immobilization (Figure 2B,C), aggregates with a rough surface can be observed. These changes are probably caused by the efficient deposition of the HRP enzyme and its further cross-linking using glutaraldehyde. Similar observations were previously described by Voss et al. [45], where horseradish peroxidase was immobilized onto porous silica nanopowder. In the case of the chitinous scaffold from *A. fistularis*, the fibers’ surface before immobilization was uniform and smooth, which confirms a well-performed isolation process (Figure 2D) [30,46,47]. By contrast, after HRP immobilization, aggregates at a size around 2–4 µm in diameter can be observed, which confirms the effective attachment of enzyme molecules onto the chitinous scaffold. As shown in Figure 2E,F, the enzymes’ aggregates fully cover the support material, which can suggest high enzyme affinity of the chitin scaffold and deposition of HRP on it. Similar observations have been described in the work of Arslan [48], who immobilized horseradish peroxidase on amine-functionalized glycidyl methacrylate-g-poly(ethylene terephthalate) fibers and used the produced biocatalytic system for removal of azo dyes from aqueous solutions. The obtained results confirm that chitinous fibers constitute a suitable matrix for enzyme immobilization. Further, it should be highly emphasized that in the presented work, the HRP enzyme was immobilized onto a chitinous scaffold isolated from *A. fistularis* marine sponge for the first time, indicating the high novelty of the presented study.

**Figure 3:**
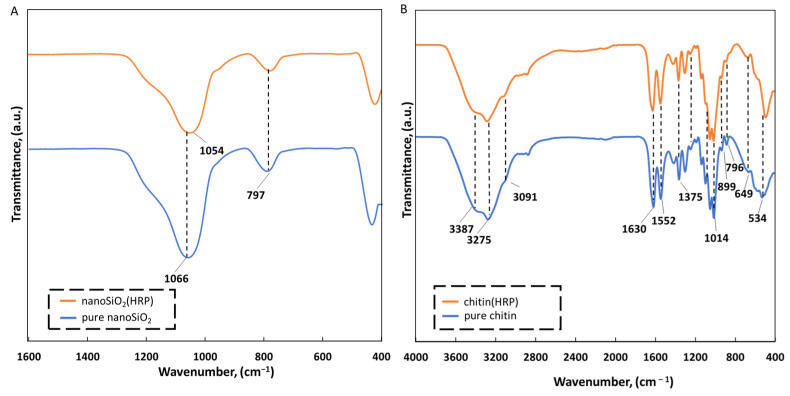
(**A**) ATR-FTIR spectra of silica nanoparticles (blue line), silica nanoparticles after HRP immobilization (orange line). (**B**) Spectra of pure chitinous scaffold (blue line), chitin after HRP immobilization (orange line).

To confirm the effective production of EE2 removal systems made of nanoSiO_2_, a chitinous scaffold and HRP, an ATR-FTIR analysis has been made (Figure 3). The most intensive band with a maximum close to 1066 cm^−1^ unambiguously corresponds to the Si–O and Si–O–Si bonds characteristic for silica [49,50,51] or Si–O–X, where X represents ethoxy groups bonded to silicon. These signals indicated the presence of stoichiometric silicon dioxide structure [17]. However, a shift of these bands toward 1054 cm^−1^ has been observed in the case of the FTIR spectrum of nanoSiO_2_ with immobilized HRP that indicates direct interactions of this group with biomolecules. A similar tendency was previously described for the approach of using cubic mesoporous silicate with horseradish peroxidase immobilized by adsorption [52]. The absorption band at 797 cm^−1^ is associated with Si–OH stretching vibration and Si–O bending vibrations of siloxane groups [52,53]. At higher wavenumbers, we observed only slight shifts, indicating the presence of hydrogen bonds (data not shown).

The infrared spectra of the pure chitinous skeleton from *A. fistularis* (blue line) was presented in Figure 3B. It was calculated that the degree of acetylation (DA) was 81%, which is typical for chitin isolated by alkali chemical treatment [44]. Thus, the degree of deacetylation (DD) was calculated as 19%; therefore, based on these results, it could be concluded that highly N-acetylated biopolymer has been obtained [36] (see Supplementary Materials). The narrow peak at wavenumber 1630 cm^−1^ corresponds to stretching vibrations of C=O bonds (amide I) in α-chitin [30,37]. Precisely, this peak corresponds to stretching vibrations from inter- (C=O· · ·H–N) and intramolecular (C=O· · ·HOCH_2_) hydrogen bonds [36]. Additionally, the presence of bands such as νN–H and νC–N at 1552 cm^−1^ correspond to α-chitin. Other bands assigned to the chitin structure were observed at 1435 cm^−1^ (CH_2_ bending and CH_3_ deformation), 1375 cm^−1^ (CH bending and CH_3_ symmetric deformation), 1150 cm^−1^ (asymmetric carbon-oxide bridge) and at 896 cm^−1^ (corresponding to glycosidic linkage and CH stretching vibrations of saccharide rings). After HRP immobilization, the spectrum of the chitin–HRP system was very similar to pure chitin, which was caused by the presence of the same functional groups onto support before and after enzyme immobilization. However, an interesting shift, from 1630 cm^−1^ to 1638 cm^−1^, was observed for the peak corresponding to amide I bonds, which could be explained by interactions between the negatively charged end of the –N–C=O group of chitin (negative charge shifted towards oxygen) and positively charged enzyme molecules, as well as being due to formation of hydrogen bonds. Moreover, the differences are visible at peaks corresponding to C—C out-of-plane bending. For the spectrum of chitin with enzyme immobilized, the peak characteristic for these vibrations was at 515 cm^−1^, whereas on the spectrum for pure chitin, it is shown at 534 cm^−1^. This shift indicates a change of enzyme microenvironment due to the creation of enzyme–support interactions [43].

### 2.2. EE2 Removal Study

After the confirmation of effective enzyme immobilization, the most efficient system for removal of EE2 was determined. Thus, the application of the following four variations of configuration of prepared EE2 removal systems for removal of estrogen have been developed to achieve complete removal of micropollutant: (i) nanoSiO_2_(HRP)–pure chitin, (ii) pure nanoSiO_2_–chitin(HRP), (iii) nanoSiO_2_(HRP)–chitin(HRP), (iv) pure nanoSiO_2_–pure chitin. This unusual combination of nanosilica, chitin from marine sponge and HRP has been used for estrogen removal for the first time. Moreover, due to the fact that process conditions are extremely important in terms of practical application of the immobilized enzymes, it was decided to investigate the effect of various process conditions, including different pH, temperature, H_2_O_2_ and EE2 concentrations on removal efficiency of EE2 from aqueous solutions using all four configurations.

**Figure 4:**
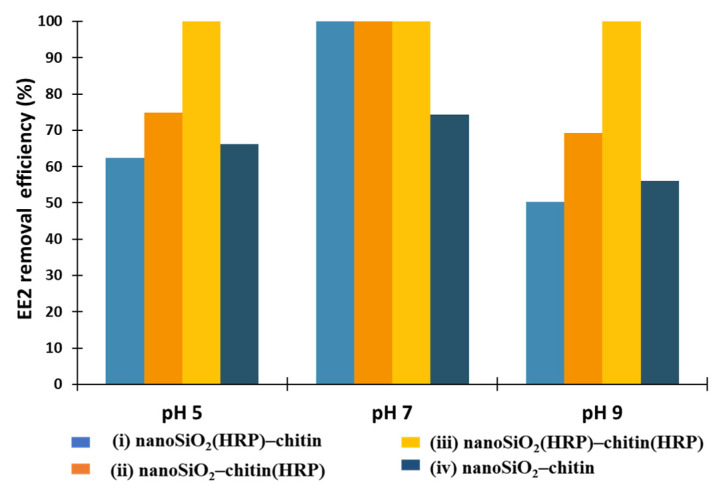
Effect of pH on removal efficiency of 17α-ethinylestradiol using prepared removal systems at various configurations (i–iv).

Oxidoreductases, such as horseradish peroxidase, were established as suitable biocatalysts for the removal of estrogens from the wastewaters [2,12]. Previous investigations regarding usability of immobilized form of HRP indicated that pH has a great importance in terms of activity of immobilized biomolecules and the removal process of estrogens [2,12,41]. The study presented by El-Nahass et al. [52] showed that pH strictly affects the activity of free and immobilized HRP due to the transport of protons in the biomolecule active site, at specific pH. The authors determined that free and immobilized enzymes onto mesoporous silicates possessed the highest activity at pH 6. However, the study presented by Xu et al. [2] showed that HRP immobilized onto Fe_3_O_4_ nanoparticles obtained the highest relative activity, at pH 7. Thus, the optimal pH for HRP activity can vary depending on the applied support material. Moreover, the removal efficiency of specific compounds, such as estrogens, could also be different, depending on the reaction environment. Thus, in the first step of this investigation, the effect of pH on the removal of EE2 were examined in pH 5, 7 and 9. As can be observed in Figure 4, the removal efficiency of EE2, ranging from 62% for the biocatalytic system (i) nanoSiO_2_(HRP)–pure chitin to 100% for system (iii), where immobilized HRP was used at both stages of EE2 removal. The highest removal efficiencies, equaling 100%, were obtained after a process carried out at pH 7 using EE2 removal systems (I, ii, and iii). In the case of (iv) system, after its application, the removal efficiency of estrogen was 74%. These results indicate that simple adsorption by nanoSiO_2_ and chitin is not sufficient enough, and catalytic action of immobilized HRP is required to attain a high removal rate. The results obtained for pH 9 in the case of the application of produced EE2 removal systems showed the lowest removal efficiencies of EE2, compared to the processes carried out at pH 5 and 7, and equaled less than 70%. However, for the biocatalytic system made of nanoSiO_2_(HRP)–chitin(HRP), the removal efficiency of estrogen was 100%. This is probably due to the fact that, in this system, more HRP biomolecules were immobilized, and the bioconversion process could be carried out more effectively, at both degradation stages. It should also be remembered that, due to the immobilization process, enzymes could be more stable under various process conditions, retaining high activity, compared to the free form of biomolecules, even at high pH conditions [42]. Despite the high bioconversion rate, it should be underlined that synergic removal of estrogen was observed by adsorption and catalytic conversion of EE2, which allowed us to obtain 100% removal efficiency, albeit with a pronounced dominance of catalytic conversion.

**Figure 5:**
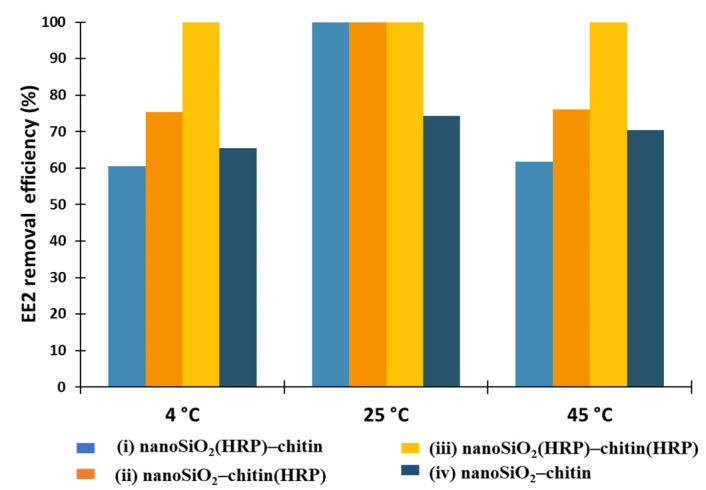
Effect of temperature on removal efficiency of 17α-ethinylestradiol using prepared removal systems at various configurations (i–iv).

In the next step, it was decided to investigate the effect of temperature on removal efficiency of EE2 using the proposed systems (Figure 5). The temperature is one of the most important parameters affecting adsorption and enzymatic conversion. As observed, at higher temperatures, biomolecules are prone to inactivation due to enzymes sensitivity and denaturation [54]. Further, it is important to lower the transformation costs in order to conduct industrial processes in ambient conditions (e.g., wastewater treatment plants). It should also be stated that both chitin and nanosilica are stable over a wide range of temperatures; therefore, they could be used as stable supports for enzyme immobilization [46,55]. In this study, EE2 removal processes were carried out at temperatures of 4, 25 and 45 °C. Previously, Xu and others [2] investigated estrone (E1) removal by horseradish peroxidase immobilized on a nanofibrous support made of poly(acrylic acid)/poly(vinyl alcohol) (PAA/PVA) with Fe_3_O_4_ nanoparticles. As observed, the highest removal efficiency of estrogen was noted at 32.4 °C, 39.4 °C, and 49.9 °C for free HRP, HRP immobilized onto PAA/PVA nanofibers, and HRP immobilized onto nanofibers with Fe_3_O_4_, respectively. Furthermore, it was observed that the removal of estrogen by immobilized biomolecules was significantly higher than after the process using free HRP at the entire investigated temperature range, which could be explained by the fact that support materials protect HRP against high-temperature-induced deactivation [56]. Surprisingly, in our research, the most suitable temperature for the most effective EE2 removal has been determined as 25 °C. In these conditions, the following three biocatalytic systems allowed us to remove 100% of EE2 from aqueous solutions: (i) nanoSiO_2_(HRP)–pure chitin, (ii) pure nanoSiO_2_–chitin(HRP), (iii) nanoSiO_2_(HRP)–chitin(HRP). For system (iv), without immobilized enzymes, adsorption of estrogen onto a nanoSiO_2_ and chitinous scaffold allowed us to obtain 74% removal efficiency. At the lowest temperature, which was 4 °C, EE2 removal efficiency ranged from 60% for removal system (i) to 100% for removal system (iii). In the case of 45 °C, the results of removal efficiencies were similar to the results obtained after process at 4 °C, which shows that the proposed removal system, irrespective of temperature value, can remove over 60% of EE2. Moreover, it was observed that after the use of biocatalytic system (iii), where HRP was immobilized onto both carriers, the removal efficiency equaled 100% for each of the investigated temperatures, suggesting a high stability of the immobilized enzyme in this temperature.

**Figure 6:**
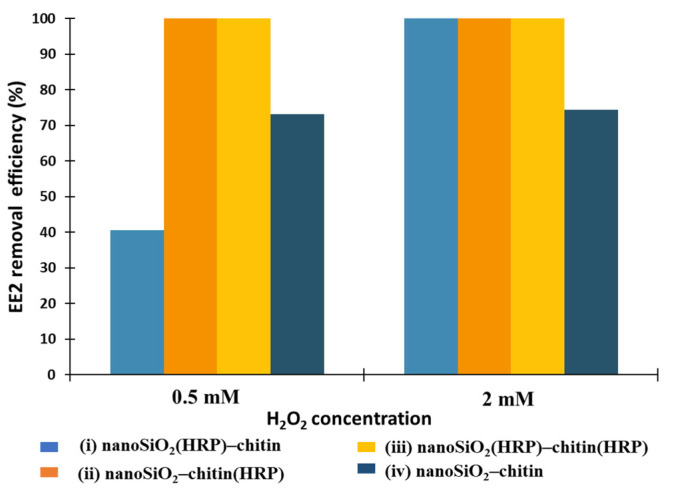
Effect of initial concentration of hydrogen peroxide (H_2_O_2_) on removal efficiency of EE2 using prepared removal systems (i–iv).

Hydrogen peroxide is usually applied as co-substrate of horseradish peroxidase. It plays the role of one-electron donor between the HRP active site and converted substrate [57]. However, too high a concentration of H_2_O_2_ in the reaction environment can negatively affect the activity of horseradish peroxidase. Wang et al. [45] used HRP immobilized onto polyacrylonitrile beads and the next such biocatalytic system was used for removal of 2,4-dichlorophenol from wastewater. The authors showed that hydrogen peroxide solution at concentration above 1 mM can inhibit the catalytic activity of biomolecules due to blocking of the enzyme’s active site. The study of Ai and co-authors [12] proved that overdose of hydrogen peroxide in conversion reactions catalyzed by HRP can act as scavengers of active radicals due to reduction reactions. The authors used s biocatalytic system composed of NH_2_-Fe_3_O_4_@SiO_2_ with HRP immobilized for degradation of estradiol (E2) from an aqueous solution. As determined, the optimal concentration of H_2_O_2_ for process catalyzed by immobilized HRP was 24 mg/L. By contrast, in another work, the optimal hydrogen peroxide concentration for estrone (E1) removal by free HRP and HRP immobilized onto PAA/PVA nanofibers was determined at 0.05 mM. Higher H_2_O_2_ concentrations negatively affected the enzymatic activity of HRP and, in consequence, a lower removal efficiency of E1 was noted. However, when support was additionally doped by Fe_3_O_4_ nanoparticles, the removal efficiency of E1 reached a maximum with the presence of H_2_O_2_ at a concentration of 0.1 mM, which could be explained by excessive H_2_O_2_ consumption by magnetite in the Fe^2+^ oxidization process. Therefore, a suitable initial concentration of hydrogen peroxide is crucial for estrogen removal by HRP-based catalytic reactions and should be determined for all support-enzyme systems. Herein, the removal of 17α-ethinylestradiol was conducted using two H_2_O_2_ concentrations, which were 0.5 and 2 mM. As shown in Figure 6, a higher concentration of H_2_O_2_ (2 mM) allowed us to fully remove EE2 from aqueous solutions using all systems containing immobilized HRP (i, ii and iii). It stays in agreement with results described by Patel et al. [58], where in situ encapsulation of horseradish peroxidase in electrospun porous silica fibers was carried out. The authors showed that enzyme encapsulated in produced fibers possessed the highest specific activity with the presence of hydrogen peroxide solution at a concentration equaling 2 mM. Moreover, a further increase in H_2_O_2_ concentration caused the inhibition of biomolecules and a decrease in its catalytic activity. In our work, it was shown that a lower concentration (0.5 mM) of hydrogen peroxide in the reaction environment caused a significant decrease in removal efficiency of EE2 to 40% using system (i), where HRP was immobilized onto nanosilica, which could be explained by limited transport of H_2_O_2_ to the active site of biomolecules immobilized onto nanoSiO_2_, cross-linked by glutaraldehyde. However, systems (ii) and (iii) allowed us to remove 100% of EE2 from aqueous solutions with the presence of hydrogen peroxide at lower concentrations. In the case of removal system (iv), where pure nanoSiO_2_ and pure chitin were used in the EE2 removal process, only adsorption as a removal mechanism was possible. The removal efficiency of EE2 was determined and equaled 73% (H_2_O_2_ concentration 0.5 mM) and 74% (H_2_O_2_ concentration 2 mM). The lack of biomolecules immobilized onto supports meant that the H_2_O_2_ concentration did not affect the removal efficiency of estrogen.

**Figure 7:**
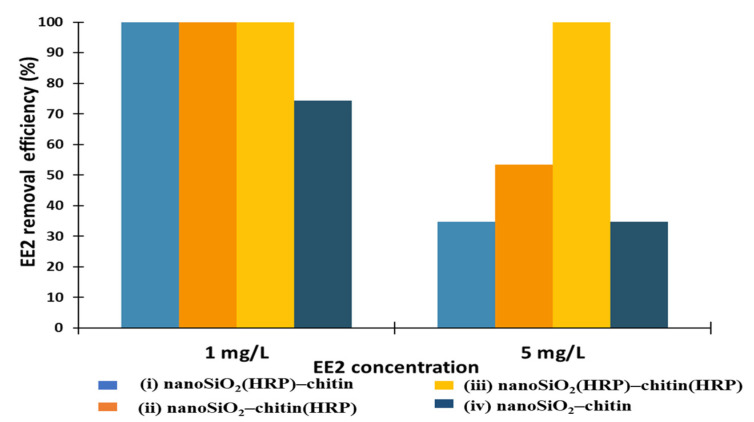
Effect of initial concentration of EE2 solution on its removal efficiency using prepared removal systems (i–iv).

17α-ethinylestradiol (EE2) is a synthetic estrogen, which is difficult to remove by conventional methods. The main sources of this compound in surface waters are livestock, hospitals, pharmacy factories and aquaculture wastewaters. A recently published article summarized EE2 concentration levels in surface waters among 32 countries around the world [59]. The authors determined that the concentration of 17α-ethinylestradiol in surface waters reaches even 33 ng/L. Moreover, the concentrations of EE2 in waters are generally higher in developing countries, compared to the developed ones [43]. Therefore, we decided to use higher concentrations (1 and 5 mg/L) of estrogen to determine the removal effectivity of this compound using prepared biocatalytic systems and to determine capability of the proposed approach for complete removal of this micropollutant (Figure 7). It was shown that EE2 was fully removed from the solution at a concentration of 1 mg/L using removal systems with immobilized HRP (i, ii and iii). However, the application of pure nanoSiO_2_ and pure chitin allowed us to remove 74% of EE2, clearly indicating adsorption of this compound onto proposed materials. The results of the removal experiments using estrogen solution at a concentration of 5 mg/L indicated that the highest removal efficiencies of estrogen (100%) were noted after the application of system (iii), where HRP was immobilized onto both supports. This could be explained by the presence of the largest amount of immobilized biomolecules on this system and, in consequence, a higher removal rate by biocatalytic action. System, where enzymes were immobilized only onto nanoSiO_2_ (i) supported by pure chitin, allowed us to remove 34% of EE2, which was similar to the result obtained after the removal process using the system without immobilized HRP (iv). Over 50% degradation efficiency of estrogen was noted after the application of system (ii), where HRP was immobilized only onto the chitinous scaffold. Thus, it might be concluded that the chitinous scaffold possesses applicability in the removal of EE2, even from solutions at high concentrations, mainly due to its 3D arrangement, easier transport of estrogen to the enzyme’s active site and simpler diffusion of bioconversion products, compared to the system based on SiO_2_.

**Figure 8:**
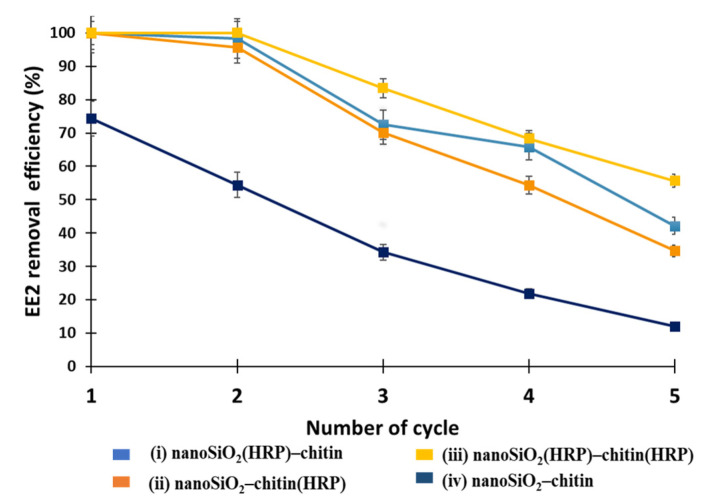
Reusability of the EE2 removal systems (i–iv) over repeated catalytic cycles.

Reusability of the proposed systems in terms of removal of estrogen from aqueous solution is one of the most important parameters affecting their practical application. In our study, it was decided to investigate reusability of the proposed systems in five consecutive catalytic cycles (Figure 8). It could be seen that the removal efficiencies of estrogen after application of each of the systems tested decreased. For example, the removal efficiency of EE2 decreased from 73% (1st catalytic cycle) to almost 12% (5th catalytic cycle) after using pure nanoSiO_2_ –pure chitin system. The probable explanation of this could be pores clogging and, in consequence, lower adsorption of EE2 onto the nanoSiO_2_ and chitinous scaffold over repeated use [60]. It was also shown that after five catalytic cycles, EE2 was removed by other systems with the following efficiencies: 42%, 35% and 56%, for (i) nanoSiO_2_(HRP)–pure chitin, (ii) pure nanoSiO_2_–chitin(HRP) and (iii) nanoSiO_2_(HRP)–chitin(HRP), respectively. The higher values of EE2 removal efficiencies were observed for system (i), compared to system (ii) in all performed cycles, which can be explained by a higher content of HRP immobilized onto nanosilica than chitin, and a more efficient enzymatic conversion. System (iii), where HRP was immobilized onto both supports, allowed us to achieve 100% removal efficiency of EE2 in the first two cycles, and then constantly decreases until 56% is reached after five cycles. The fact could be affected by HRP enzyme consumption, its inactivation over repeated use, by blocking of active sites by conversion products and partial elution of biomolecules from the support [11]. Nevertheless, the removal of over 50% of EE2 after five degradation steps shows formation of stable enzyme–support interactions, i.e., an improvement of enzyme stability over repeated use.

### 2.3. Mechanism of 17α-Ethinylestradiol Removal

**Figure 9:**
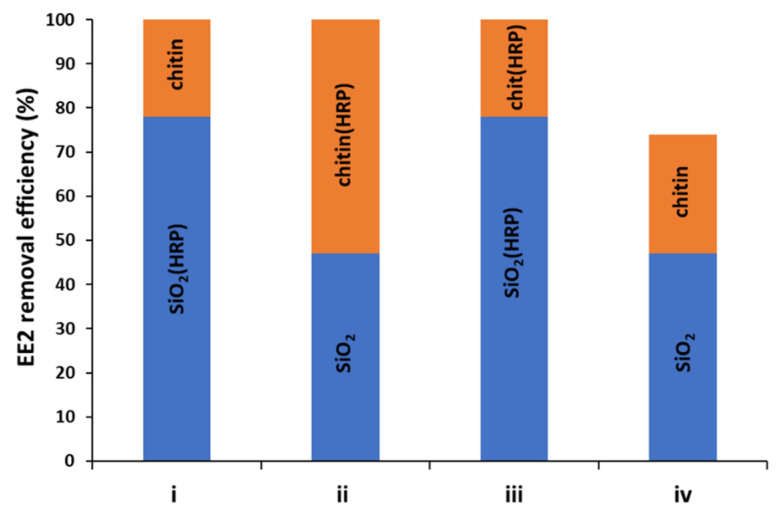
Synergistic mechanism and the contribution of each factor in overall removal rate of 17α-ethinylestradiol by systems (i–iv) proposed in the study.

After confirmation of effective enzyme immobilization and determination of the most suitable conditions for EE2 removal, it was also important to determine the mechanism of estrogen removal. In the study, the following four combinations of prepared systems have been developed: (i) nanoSiO_2_(HRP)–pure chitin, (ii) pure nanoSiO_2_–chitin(HRP), (iii) nanoSiO_2_(HRP)–chitin(HRP), (iv) pure nanoSiO_2_–pure chitin (Figure 9). Due to the fact that, in the removal process, materials with and without immobilized HRP were used, it is expected that the mechanism of EE2 removal varies for each of the systems, and the percentage contribution of adsorption and enzymatic conversion will be different. Experiments with pristine nanosilica and chitin showed that these materials are capable of adsorption of 48% and 27% of 17α-ethinylestradiol, respectively. Although both materials used in the study are known for their good sorption properties, the chemical structure and chemical moieties of the 17α-ethinylestradiol limit its effective adsorption [61] and makes single adsorption unsuitable for removal of this micropollutant. This is proved by the result of an adsorption experiment using pristine materials, which showed that after two stages of EE2 sorption, less than 75% of the pollutants were removed from the solution. As expected, the adsorption capacity of materials with immobilized HRP subjected to thermal inactivation were lower, as compared to pristine materials. Due to the deposition of the enzyme molecules and saturation of the surface-active centers, the adsorption rate of EE2 onto nanosilica and chitin attained 40% and 22%, respectively. By contrast, nanosilica and chitin with active immobilized HRP showed significantly higher removal of EE2, which reached 78% and 63%, respectively. This data clearly shows that in the systems with immobilized HRP, the removal of EE2 occurred due to simultaneous adsorption and biocatalytic conversion. Nevertheless, the application of a single system with immobilized enzymes makes it impossible to completely remove EE2, and use of a supporting stage is required to achieve a higher removal rate. From the presented results, it is also clear that the percentage contribution of catalytic conversion in degradation of EE2 plays an important role and increases its removal rate by about 40% for both analyzed systems. However, in general consideration of the removal mechanism, it could be summarized that, in all tested systems, adsorption is a dominant mechanism, whereas catalytic conversion supports this process. Finally, based on the obtained data, it should be highlighted that nanosilica seems to be a more promising material for adsorption/catalytic conversion of 17α-ethinylestradiol, because systems made of silica and/or silica with immobilized HRP made it possible to completely remove EE2 from the solution, whereas use of chitin-based systems allowed for around a 90% removal rate of EE2.

Therefore, based on the presented data, it is clear that 100% removal of EE2 by three tested biocatalytic system is possible only due to the simultaneous action of adsorption and enzymatic conversion. In the systems with immobilized HRP, oestrogen is partially adsorbed onto the support material, which, besides direct removal of micropollutants, also ensures the supply of fresh substrate for the enzymes that facilitate catalytic conversion of oestrogen and increase its removal rate. Nevertheless, in each of the systems with 100% degradation efficiency, the first stage of the removal has to be supported by adsorption or enzymatic conversion on the second stage. This data clearly indicates the necessity to design and develop systems for combined degradation of micropollutants based on simultaneous adsorption and biocatalytic conversion to achieve high removal rate of hazardous compounds. Furthermore, the collected results also indicate the presented systems as promising tools for wastewater treatment. We have also proposed the pathways of catalytic conversion of EE2 (Figure S2); however, a detailed examination of the products of catalytic conversion would also be required in order to clearly define the composition of the post-reaction mixture.

## 3. Materials and Methods

### 3.1. Chemicals and Materials

Air-dried specimens of the marine sponge *A. fistularis* (Figure 1) have been delivered by INTIB GmbH (Freiberg, Germany). The chemical reagents used for isolation of sponge chitinous skeleton—sodium hydroxide and acetic acid—were supplied by Chempur (Poland). Silica in form of nanopowder (>99.8%, BET: 225 m^2^/g), horseradish peroxidase (EC 1.11.1.7, activity ~150 U/mg), 50 mM phosphate buffer (pH 7–9), 100 mM acetate buffer (pH 3–6), bis–trimethylsilyltrifluoroacetamide (BSTFA) + 1% trimethylsilyl chloride (TMCS), 17α-ethinylestradiol (≥99%) (EE2), and Bradford reagent were purchased from Sigma Aldrich (Saint Louis, MO, USA).

### 3.2. Chitinous Scaffold Isolation from A. fistularis Marine Sponge

A chitin-based skeleton was obtained by acid-base treatment of air-dried specimens of *A. fistularis* marine sponge, with a total isolation time of 6 days. Proposed method is analogous to previously published article [30]. Isolation processes start by treatment fragments of sponge into the deionized water for 4 h to remove of water-soluble impurities. Then, the skeleton was treated by 2.5 M NaOH solution as decellularization and deproteinization agent for 2 days at 36 °C. Next, partially deproteinized skeleton was neutralized with deionized water several times and immersed for 5 h into 20% acetic acid for demineralization. The following procedure was replicated until pure chitin was obtained. Finally, transparent chitinous scaffolds were neutralized with deionized water and then kept in 70% ethanol at 4 °C for further actions.

### 3.3. Horseradish Peroxidase Immobilization onto Chitin and Nanosilica and Amount of Immobilized Enzyme

Before horseradish peroxidase immobilization, the chitin-based scaffolds were cut into 5 × 8 mm pieces (~50 mg). Then, samples were placed separately into a beaker containing 2 mL of HRP solution in phosphate buffer at pH 7 and concentration 1 mg/mL. The vials were placed in an incubator (IKA Werke GmbH, Staufen im Breisgau, Germany) and shaken at 200 rpm for 3 h at 25 °C. After the immobilization, samples were moved out from the solution, washed several times with phosphate buffer at pH 7 to remove unbounded HRP, and used in further tests. In the case of horseradish peroxidase immobilization onto nanosilica, 50 mg of powder was placed in a vial with 2 mL of HRP solution in phosphate buffer (as described above). After shaking (200 rpm, 3 h, 25 °C) samples have been centrifuged (12,000 rpm) and supernatant has been decanted from the precipitant. Then, 2 mL of 0.1% glutaraldehyde solution (pH 7) was added to the silica with HRP immobilized and mixed for 1 h (200 rpm, 25 °C). After this time, the obtained system was centrifuged (12,000 rpm) and washed several times using phosphate buffer. Immobilization process was performed at pH 7, which is the most favorable by HRP enzyme due to irregular displacement of hydrogen cations in the environment across the biomolecule [11]. Moreover, neutral pH allows free interactions between negatively charged end of –N–C=O group of chitin (negative charge shifted towards oxygen) and positively charged enzyme. These kinds of forces may further improve stability of the biomolecule structure [11,62].

In both cases, to determine immobilization efficiency, supernatant of HRP solution after immobilization, as well as buffer solutions used for washing of systems after immobilization, were analyzed by spectrophotometric measurements using Bradford method [63]. Bradford reagent was mixed in a 1:1 volumetric ratio with analyzed solution. After 5 min of incubation, absorbance was measured at 595 nm. The enzyme concentrations were determined using bovine serum albumin standard curve. The immobilization efficiency (%) was calculated using the following formula by considering initial amount of the enzyme and amount of the HRP in the supernatant after immobilization and in the solutions after biosystem washing (Equation (1)):

(1) $$Immobilization\;efficiency\;\left(\%\right)=\frac{C_{R}+C_{W}}{C_{I}}\cdot 100\%$$

where *C_R_*, *C_W_* and *C_I_* denotes amount of the enzyme in the supernatant after immobilization, amount of the enzyme in the solutions after washing and initial amount of the enzyme.

### 3.4. Removal of EE2 by Simultaneous Adsorption and Catalytic Conversion

**Figure 10:**
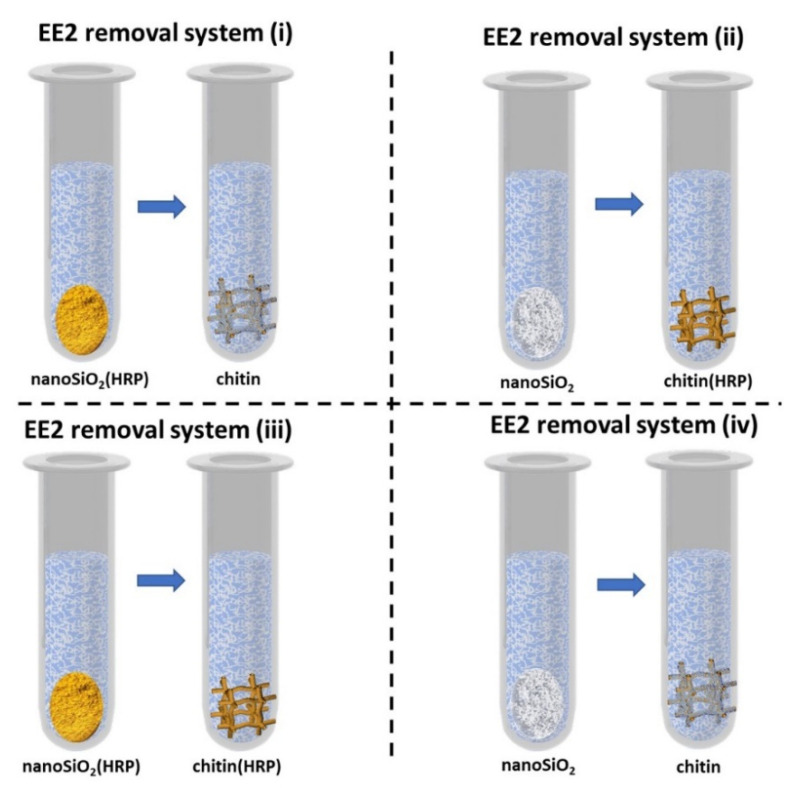
Schematic representation of EE2 removal systems (i–iv) investigated in the study.

In order to investigate the removal efficiency of EE2 by simultaneous adsorption and catalytic conversion, the support with HRP immobilized were used in various configurations, each consisting of two stages, as follows: (i) nanoSiO_2_(HRP)–pure chitin, (ii) pure nanoSiO_2_–chitin(HRP) and (iii) nanoSiO_2_(HRP)–chitin(HRP). System (iv), containing pristine nanosilica and pristine chitin, was applied for comparison (Figure 10). In each of the applied approaches, in the first stage, estrogen removal was carried out using first system and then supernatant was transferred to the second stage system. The duration of each stage was 24 h (48 h for whole degradation process), whereas other parameters varied between processes. To examine the effect of pH, EE2 solutions were prepared at specific buffer solutions at pH values ranging from 3 to 9. The reactions were carried out at 25 °C using EE2 solution at concentrations 1 mg/L and 2 mM H_2_O_2_. Hydrogen peroxide plays role of one-electron donor between HRP active site and converted substrate. To determine the effect of temperature, the removal processes of EE2 were carried out at temperatures 4 °C, 25 °C and 45 °C, using EE2 solution at pH 7, and concentrations of 1 mg/L and 2 mM H_2_O_2_. The effect of estrogen concentration on its removal efficiency was determined at 25 °C using solutions at pH 7 and concentrations 1.0 and 5 mg/L and with the presence of 2 mM H_2_O_2_. Moreover, the effect of H_2_O_2_ concentration was determined using 0.5 mM and 2 mM of hydrogen peroxide, at 25 °C, using EE2 solutions at pH 7 and concentration 1 mg/L. After each stage, the system was separated from reaction mixture by centrifugation and transferred to the fresh EE2 solution for second stage removal. Samples after estrogen removal by adsorption were centrifuged and subjected to GC-MS analysis.

To determine removal efficiency of EE2 by adsorption using pristine silica and pristine chitin, 50 mg of the nanoSiO_2_ and 50 mg of dry chitin-based scaffold were weighed out and then placed in separate vials, containing 2 mL of the EE2 solution. The process was carried out for 24 h (in process conditions as above). Similar experiments were performed to determine sorption properties of the support materials with thermally inactivated enzyme. Briefly, nanoSiO_2_ and chitin with immobilized HRP were subject for thermal inactivation (5 h, 80 °C) and then placed in 2 mL of EE2 solution for 24 h. Samples after estrogen removal by adsorption were centrifuged and subjected to GC-MS analysis. The experiments on the removal of EE2 were performed in duplicate and the results presented in each of the graph are presented as a mean value, which standard deviation does not exceed 5%.

### 3.5. Reusability of the Proposed EE2 Removal Systems

The reusability of the proposed systems was investigated over 5 consecutive catalytic cycles. Each catalytic cycle means the removal of EE2 carried out for 48 h (24 h for single stage for each support with or without HRP immobilized), at pH 7 and temperature 25 °C, with EE2 solution at concentration 1 mg/L and with the presence of 2 mM H_2_O_2_. After each catalytic cycle, the obtained removal system was removed from the mixture, washed carefully with phosphate buffer at pH 7 and placed into a new EE2 solution at concentration 1 mg/L.

### 3.6. Characterization Techniques

The materials nanoSiO_2_ and chitinous scaffold were visualized by advanced digital microscope Keyence VHX-7000 digital (Osaka, Japan) stocked with VH-Z20R swing-head zoom lenses (maximal magnification 200×). The ATR–FTIR infrared spectroscopy analysis was used for the characterization prepared materials with and without HRP immobilized. The presence of characteristic signals for recorded functional groups were detected using a VERTEX 70 spectrometer (Bruker, Karlsruhe, Germany). The wide wavenumber range of 4000–500 cm^−1^ (resolution 0.5 cm^−1^) spectra was recorded. The samples were observed using a scanning electron microscope (SEM) Mira 3 (Tescan, Brno, Czech Republic) equipped with EDS Ultim Max 65 (Oxford Instruments, High Wycombe, UK). An accelerating voltage of 12 kV has been applied. A thin gold layer coating with a thickness of approximately 20 nm was deposited on each sample. The amount of HRP immobilized was examined using spectrophotometric measurements by V750 UV-Vis spectrophotometer (Jasco, Tokyo, Japan). The measurements were performed at 595 nm according to the Bradford method. The quantitative analysis of the EE2 in samples were evaluated using gas chromatography coupled with mass spectrometry. The methods to be used was planned according to Gunatilake et al. [64] with some modifications. Briefly, 2 mL of liquid after degradation process was placed to Eppendorf tubes and dried out for 24 h at 45 °C (Eppendorf^®^ Concentrator Plus, Darmstadt, Germany) and then mixed with 0.1 mL of BSTFA + 1% TMCS. Thereafter, the samples were analyzed using Pegasus 4D GCxGC-TOFMS (Leco Corp., St. Joseph, MI, USA) supplied with chromatographic column BPX5 (30 m × 250 µm × 0.25 µm) produced by SGE Analytical Science Europe Ltd. (Milton Keynes, UK). The total flow was 1.5 mL/min and the oven temperature was programmed as follows: 80 °C, increase 60 °C/min up to 260 °C, increase 10 °C/min, maintained for 4 min, increase 40 °C/min up to 300 °C. The transfer line and ion source temperatures were 170 °C and 250 °C, respectively. The electron energy in ion source was −70 V. The concentration was calculated based on standard curve prepared for samples of known EE2 concentration. The error value in each of the experiments (based on the mean and standard deviation from three experiments) does not exceed 5%.

## 4. Conclusions

In the presented work, a novel approach for the removal of 17α-ethinylestradiol from water solutions using simultaneous adsorption and biocatalytic conversion was shown. This approach consists of the application of a two-stage process using horseradish peroxidase immobilized onto nanosilica or a 3D fibrous chitinous scaffold from *A. fistularis* marine sponges, supported by the use of pristine materials or materials with immobilized enzymes in the second stage of the removal. Among the tested systems, the approach concerning the use of nanosilica-immobilized HRP in the first stage, and chitin-immobilized HRP in the second stage, allowed 100% removal of EE2 over a wide range of pH and temperature conditions, even from an estrogen solution at a concentration of 5 mg/L. Moreover, this system showed great reusability, as it facilitates the removal of around 60% of micropollutants after five consecutive reaction cycles. Finally, a study on the mechanism of EE2 removal showed that high degradation efficiencies were attained only due to the synergistic action of adsorption and horseradish peroxidase-supported enzymatic conversion. The described approach is eco-friendly and is a promising alternative to the removal of micropollutants from wastewater, including dyes, pesticides or pharmaceuticals; however, future studies are still highly required to support the transfer of the presented solutions into a larger scale.

## Data Availability

Not applicable.

## Supplementary Materials

The following are available online: Figure S1: Silica nanopowder (A) before and (B) after HRP enzyme immobilization. Chitinous scaffold from *A. fistularis* marine sponge (C) before and (D) after HRP immobilization. Figure S2. The general scheme of possible enzymatic conversion reaction.

## Sample Availability

Samples of the compounds are available from the authors after request.
