# Supplementary material for: Biocatalytic System Made of 3D Chitin, Silica Nanopowder and Horseradish Peroxidase for the Removal of 17α-Ethinylestradiol: Determination of Process Efficiency and Degradation Mechanism
Source: Molecules. 2022 Feb 17;27(4):1354. doi: 10.3390/molecules27041354 (PMC8876220; doi:10.3390/molecules27041354)
Supplement: Supplementary file 1 [file molecules-27-01354-s001.zip › molecules-1599158-supplementary.pdf]

molecules

Supplementary Materials

Biocatalytic system made of 3D chitin, silica nanopowder and horseradish peroxidase for the removal of 17α-ethinylestradiol: Determination of process efficiency and degradation mechanism

Tomasz Machałowski [1], Katarzyna Jankowska [1,2], Karolina Bachosz [1], Wojciech Smułek [1], Hermann Ehrlich[3,4], Ewa Kaczorek [1], Jakub Zdarta [1,*], Teofil Jesionowski [1,*]

[1] Institute of Chemical Technology and Engineering, Faculty of Chemical Technology, Poznan University of Technology, Berdychowo 4, 60-965 Poznan, Poland; tomasz.g.machalowski@doctorate.put.poznan.pl (T.M); karolina.h.bachosz@doctorate.put.poznan.pl (K.B); wojciech.smulek@put.poznan.pl (W.S); ewa.kaczorek@put.poznan.pl (E.K);

[2] Process and Systems Engineering Centre (PROSYS), Department of Chemical and Biochemical Engineering, Technical University of Denmark, Søltofts Plads, Building 227, DK-2800 Kongens Lyngby, Denmark; katjan@kt.dtu.dk (K.J)

[3] Institute of Electronic and Sensor Materials, TU Bergakademie Freiberg, Gustav-Zeuner str. 3, 09599 Freiberg, Germany; (hermann.ehrlich@esm.tu-freiberg.de (H.E)

[4] Center for Advanced Technologies, Adam Mickiewicz University, Uniwersytetu Poznanskiego 10, 61-614 Poznan, Poland

* Correspondence: Teofil Jesionowski (T.J); e-mail: teofil.jesionowski@put.poznan.pl, Jakub Zdarta (J.Z); e-mail: jakub.zdarta@put.poznan.pl

Citation: Machałowski, T.; Jankowska, K.; Bachosz, K.; Smułek, W.; Ehrlich, H.; Kaczorek, E.; Zdarta, J.; Jesionowski, T. Biocatalytic System Made of 3D Chitin, Silica Nanopowder and Horseradish Peroxidase for the Removal of 17α-Ethinylestradiol: Determination of Process Efficiency and Degradation Mechanism. *Molecules* **2022**, *27*, 1354. https://doi.org/10.3390/molecules27041354

Academic Editors: Marek Kosmulski, Zbigniew Adamczyk and Piotr Warszyński

Received: 30 January 2022

Accepted: 15 February 2022

Published: 17 February 2022

Publisher's Note: MDPI stays neutral with regard to jurisdictional claims in published maps and institutional affiliations.

Copyright: © 2022 by the authors. Licensee MDPI, Basel, Switzerland. This article is an open access article distributed under the terms and conditions of the Creative Commons Attribution (CC BY) license (https://creativecommons.org/licenses/by/4.0/).

1. Methods

1.1. Determination of DA and DD by Attenuated Total Reflectance–Fourier Transformation Infrared Spectroscopy (ATR-FTIR)

The degree of acetylation (DA) as follows—Equation (1) and the degree of deacetylation (DD) using Equation (2) were calculated for isolated chitinous scaffold[1]:

$$DA\% = [(A_{1654}/A_{3432})\cdot 100\%)]/1.33 \quad (1)$$

$$DD\% = 100\% - DA\% \quad (2)$$

where, (A_{1654}/A_{3432}) - is absorbance at 1654 cm^{-1} and 3432 cm^{-1}, respectively

2. Results

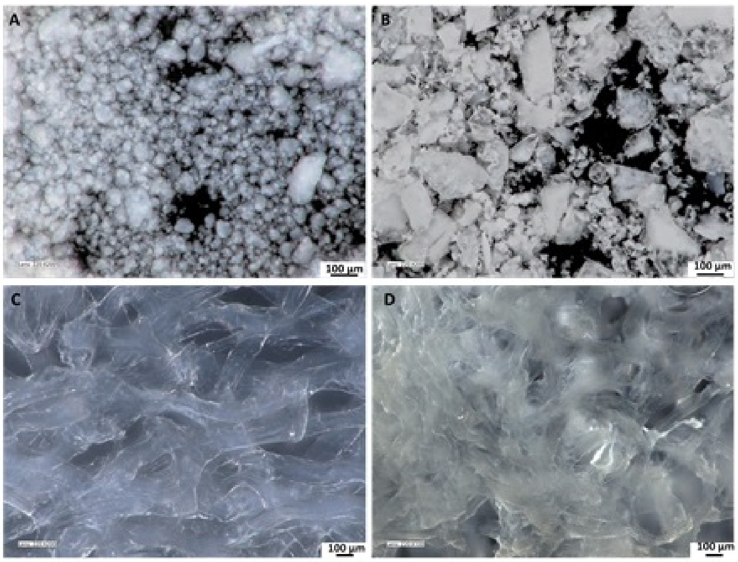

Figure S1. Silica nanopowder (A) before and (B) after HRP enzyme immobilization. Chitinous scaffold from *A. fistularis* marine sponge (C) before and (D) after HRP immobilization.

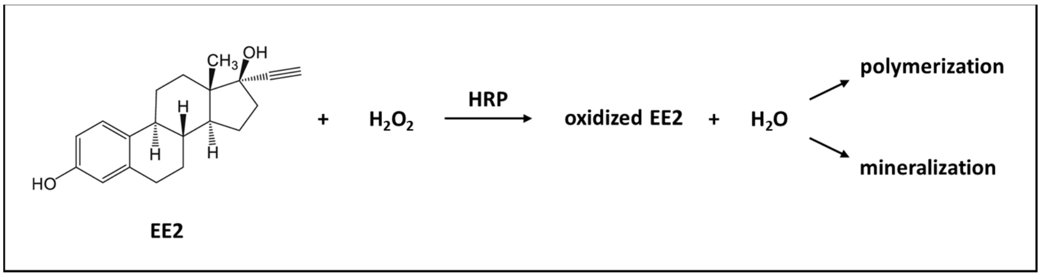

Figure S2. The general scheme of possible enzymatic conversion reaction.

References

1. Gbenebor, O.P.; Adeosun, S.O.; Lawal, G.I.; Jun, S.; Olaleye, S.A. Acetylation, crystalline and morphological properties of structural polysaccharide from shrimp exoskeleton. *Eng. Sci. Technol. an Int. J.* **2017**, *20*, 1155–1165.
